# Prevalence of cognitive impairment and its association with clinical variables among Patients with Mood Disorders

**DOI:** 10.1192/j.eurpsy.2022.2246

**Published:** 2022-09-01

**Authors:** S. Nahar, M. Khan

**Affiliations:** 1 National Institute of Mental Health, Psychiatry, Dhaka South, Bangladesh; 2 Sohrawardy Medical College Hospital, Psychiatry, Dhaka, Bangladesh

**Keywords:** Mood disorders, bipolar disorders, COGNITIVE, depressive disorders

## Abstract

**Introduction:**

*Mood disorders are common psychiatric illnesses with major disability and mortality and it is estimated that 8% to 20% of the population experience a depressive episode at some point in their lives.*

**Objectives:**

*To find out the prevalence of cognitive impairment among patients with Mood Disorders i.e, Major Depressive Disorder (MDD) and Bipolar Mood Disorder (BMD), etc. and to find out the status of cognitive impairment with clinical variables of Mood Disorders.*

**Methods:**

*This was a descriptive cross-sectional study conducted among the patients attending both the inpatient and outpatient departments of the National Institute of Mental Health, Dhaka. The duration of the study was fourteen months starting from July 2011 to September 2012. A total of one hundred and thirty-three (n=133) patients who fulfilled the inclusion-exclusion criteria were selected.*

**Results:**

*The mean age of onset of mood disorder was 30.1± 10.7years.60.2% were male and 39.8% were female respondents. Cognitive impairment was found among 43.6% of the respondents. A substantial proportion of the study population was found to have cognitive impairment. In this study, the cognitive status of the respondents was not associated with the duration of illness (p>0.5).*

**Conclusions:**

*So assessment of cognitive status should be an essential part of the management of this group of people.*

**Disclosure:**

No significant relationships.

